# Complement C4 Prevents Viral Infection through Capsid Inactivation

**DOI:** 10.1016/j.chom.2019.02.016

**Published:** 2019-04-10

**Authors:** Maria Bottermann, Stian Foss, Sarah L. Caddy, Dean Clift, Laurens M. van Tienen, Marina Vaysburd, James Cruickshank, Kevin O’Connell, Jessica Clark, Keith Mayes, Katie Higginson, Heidrun E. Lode, Martin B. McAdam, Inger Sandlie, Jan Terje Andersen, Leo C. James

**Affiliations:** 1 Protein and Nucleic Acid Chemistry Division, Medical Research Council, Laboratory of Molecular Biology, Cambridge CB2 0QH, UK; 2 Centre for Immune Regulation (CIR) and Department of Biosciences, University of Oslo, Oslo N-0316, Norway; 3 CIR and Department of Immunology, Rikshospitalet, Oslo University Hospital, Oslo N-0372, Norway; 4 Department of Pharmacology, Institute of Clinical Medicine, University of Oslo and Oslo University Hospital, Oslo N-0372, Norway

**Keywords:** complement, adenovirus, host-pathogen, gene therapy, non-enveloped virus, complement-mediated neutralization, humoral immunity, complement C4, TRIM21, neutralizing antibodies

## Abstract

The complement system is vital for anti-microbial defense. In the classical pathway, pathogen-bound antibody recruits the C1 complex (C1qC1r_2_C1s_2_) that initiates a cleavage cascade involving C2, C3, C4, and C5 and triggering microbial clearance. We demonstrate a C4-dependent antiviral mechanism that is independent of downstream complement components. C4 inhibits human adenovirus infection by directly inactivating the virus capsid. Rapid C4 activation and capsid deposition of cleaved C4b are catalyzed by antibodies via the classical pathway. Capsid-deposited C4b neutralizes infection independent of C2 and C3 but requires C1q antibody engagement. C4b inhibits capsid disassembly, preventing endosomal escape and cytosolic access. C4-deficient mice exhibit heightened viral burdens. Additionally, complement synergizes with the Fc receptor TRIM21 to block transduction by an adenovirus gene therapy vector but is partially restored by Fab virus shielding. These results suggest that the complement system could be altered to prevent virus infection and enhance virus gene therapy efficacy.

## Highlights

• Complement components C1 and C4 mediate potent neutralization of adenovirus
• Deposition of C4b on the viral capsid inactivates capsid disassembly
• C4 exerts direct antiviral functions independent from its role as a C3-convertase
• C4 antiviral functions synergize with TRIM21-mediated virus neutralization

## Graphical Abstract

**Figure:**
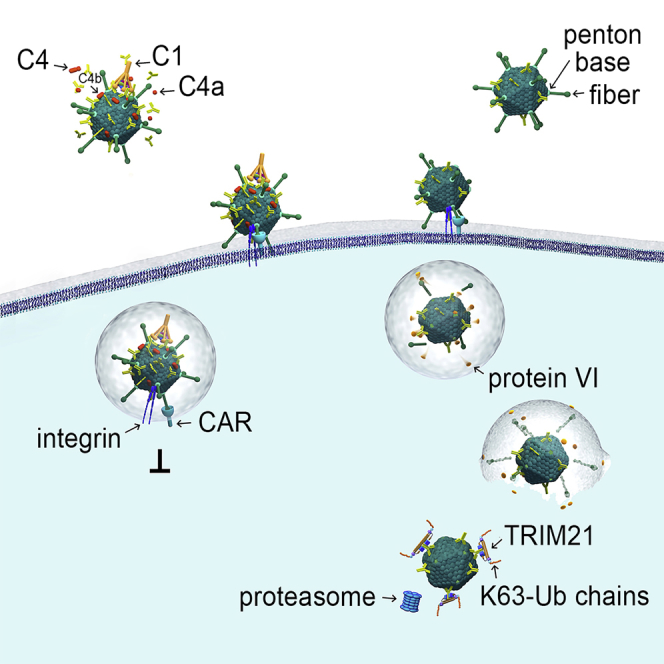

## Teaser

The complement system is pivotal to the humoral immune response. Bottermann et al. demonstrate that complement proteins C1 and C4 possess potent antiviral activity independent of downstream components. C4b is deposited on the viral capsid, preventing capsid disassembly and virus entry into the cytosol, a prerequisite for productive virus infection.

## Introduction

The complement system encompasses >20 serum proteins that attack circulating pathogens, labeling them for destruction and promoting an inflammatory immune response. Complement can label pathogens in three ways, referred to as the alternative, mannose-binding lectin, and classical pathways. Of the three, the classical pathway is the most broadly effective, as it uses anti-pathogen antibodies and thus has the potential not only to recognize any target but also to direct complement to a specific pathogen during infection.

There are two key properties that make the complement system so efficient at coating pathogens. First, it functions as an enzymatic cascade where preceding components catalyze the accumulation of subsequent proteins, thereby amplifying the response. Second, complement components couple themselves to a surface covalently, meaning that association with a pathogen is thermodynamically irreversible and long lived. At the core of the complement cascade are three paralogous proteins, C3, C4, and C5—the former two of which undergo covalent attachment. Under the classical pathway, pathogen-bound IgM or IgG first recruits the C1 complex (C1qC1r_2_C1s_2_), which cleaves C4 to expose its thioester and drive pathogen coupling. Cleaved C4b forms a complex with C2a called the “C3 convertase,” which proteolyzes C3, exposing its thioester and catalyzing surface deposition and resulting in C3b opsonization.

Since C3b opsonization is vital for downstream processes such as membrane-attack-complex formation and phagocytosis, C3 is the most extensively studied complement component. In contrast, C4 has been studied almost exclusively in its context as a convertase for C3, and any distinct mechanisms that it may have remain largely unexplored. Partly, this is because as an upstream component, defects to C4 may have a knockon effect on C3. However, human genetics suggest that C4 has important immune roles unrelated to its C3 convertase function. Approximately 75% of patients with C4 deficiency have systemic lupus erythematosus (SLE), whereas <10% of those deficient in C3 have lupus-like symptoms (Carneiro-Sampaio et al., 2008).

The importance of C4 in immunity can also be inferred from viral mechanisms of antagonism. Vaccinia complement-control protein mimics C4-binding protein, thereby accelerating C4 removal from the viral surface (McKenzie et al., 1992), while flavivirus NS1 protein binds C4 and recruits C1s, causing C4 cleavage in solution and thus reducing surface deposition (Avirutnan et al., 2010). Complement neutralization has so far only been demonstrated for enveloped viruses, with C4 exerting neutralization activity independently from C3 in the cases of equine arteritis virus (EAV) and herpes simplex virus 1 (HSV-1) (Cooper and Nemerow, 1983). However, a recent study found that Factor X was required to promote adenovirus infection in C4-competent as well as C3-deficient mice, suggesting the presence of an unknown C4-dependent mechanism that blocks infection of non-enveloped viruses (Xu et al., 2013).

Here, we describe a C4-dependent antiviral mechanism that is independent of all downstream complement components. We show that C4 deposition inactivates the capsid of the model non-enveloped virus human adenovirus 5 (Ad5) by interfering with the key capsid disassembly processes of fiber shedding and protein VI exposure, preventing it from entering the cell cytosol and thus blocking infection.

## Results

### Complement Components C1 and C4 Mediate Potent Antibody-Dependent Neutralization of Ad5

**Figure 1:**
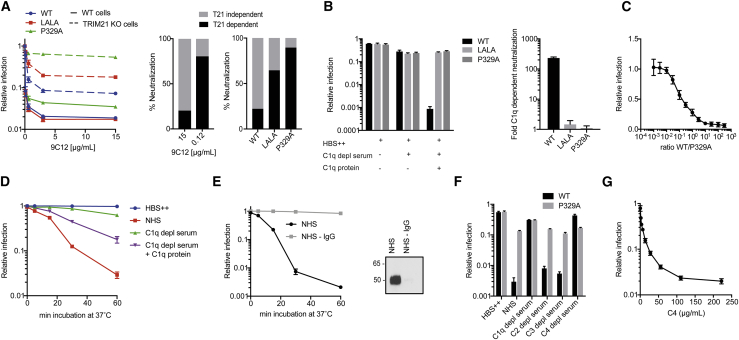
Complement Components C1 and C4 Mediate Potent Antibody-Dependent Neutralization of Ad5 (A) Neutralization of Ad5 in 293T-WT and TRIM21 KO cells using 9C12-WT and mutants LALA and P329A (left). The percentage of TRIM21-dependent and -independent neutralization using 9C12-WT at 15 μg/mL and 0.12 μg/mL (middle), as well as the percentage of TRIM21-dependent and -independent neutralization using each antibody at 15 μg/mL (right), is depicted. (B) Relative infection (left) and C1q dependent neutralization (right) in HeLa TRIM21 KO cells using 9C12-WT, LALA, or P329A and the indicated serum. (C) Neutralization of Ad5 in HeLa TRIM21 KO cells using different ratios of 9C12-WT and P329A. (D) Neutralization of Ad5 in HeLa TRIM21 KO cells. Ad5 was incubated with the indicated serum for the depicted amount of time. (E) Neutralization of Ad5 in HeLa TRIM21 KO cells. Ad5 was incubated with NHS or NHS depleted of IgG for the depicted amount of time (left). Western blot showing IgG depletion of NHS (right). Original western blots are included in Figure S6. (F) Neutralization of Ad5 in HeLa TRIM21 KO cells in the presence of human serum depleted of different complement components using 9C12-WT and mutant P329A. (G) Neutralization of Ad5 in the presence of 9C12-WT using 200 ng/mL C1 and the indicated concentrations of C4. Error bars depict the mean ± SEM of nine replicates acquired in three independent experiments (B, C, F, and G); mean ± SEM of six replicates acquired in two independent experiments (A and D); mean ± SD of three replicates acquired in one representative experiment (E).

To determine whether there are undescribed antibody-dependent antiviral mechanisms, we carried out adenovirus infection experiments in which we ablated known antibody receptor interactions. Using a recombinant mouse-human chimeric monoclonal antibody (mAb) against the main coat protein and primary immunogen of Ad5 (hexon), called 9C12 (9C12-WT), we introduced mutations L234A/L235A (LALA) to prevent Fc gamma receptor (FcγR) binding (Wines et al., 2000) and P329A to prevent C1q interaction (Idusogie et al., 2000). We performed matched antibody titrations during adenoviral challenge of either wild-type (WT) cells or cells deficient of the intracellular antibody receptor TRIM21 (Figure S1A). TRIM21 detects antibody-bound viruses that have entered the cytosol by binding the IgG Fc region with its PRYSPRY domain (Mallery et al., 2010). It then becomes activated and undergoes autoubiquitination, which results in recruitment of the proteasome and degradation of the viral particle (Fletcher et al., 2014). It is an important component of antibody-mediated protection against viruses such as Ad5 (Bottermann et al., 2018). Interestingly, while both anti-Ad5 antibody mutants only minimally affected the persistent fraction of infected WT cells, they strongly reduced neutralization in TRIM21 knockout (KO) cells, P329A more notably so than LALA (Figure 1A, left). TRIM21-independent neutralization was especially prominent at a high antibody concentration (Figure 1A, middle), and use of the P329A mutant largely converted this neutralization from a TRIM21-independent effect to a TRIM21-dependent effect (Figure 1A, right). 293Ts and HeLas do not express FcγRs (Figures S1B–S1D); however, the LALA mutation also affects binding to C1q (Wang et al., 2018), suggesting that this mutant may be ablating neutralization because of reduced C1q recruitment, similar to P329A (Figure S1E).

To confirm that C1q mediates neutralization of adenovirus, we carried out experiments in TRIM21 KO cells, which allowed us to exclusively assess TRIM21-independent neutralization. Crucially, we observed little neutralization using either buffer (HBS++) or C1q-depleted serum (Figure 1B). However, reconstitution of C1q-depleted serum with C1q protein resulted in potent Ad5 neutralization with the WT antibody, while the LALA and P329A mutants were largely inactive, thereby underlining a role for C1q in Ad5 infection. We investigated the threshold for antibody activation of C1q by determining how many antibody molecules per virus are necessary for efficient C1q-dependent neutralization. To this end, we performed a neutralization assay with normal human serum (NHS) using different ratios of WT:P329A while keeping the total antibody concentration >160 nM (Figure 1C). Using this approach, we determined the EC_50_ of the WT/P329A ratio to be 0.14 (SEM ± 0.01). Since we previously demonstrated that at saturating concentration (>100 nM) approximately 205 9C12 molecules bind to each Ad5 virion (McEwan et al., 2012), we established the number of WT antibodies per virus at the EC_50_ as 25.1 (SEM ± 1.7). Notably, however, persistent fraction is only reached when WT outcompetes P329A 10-fold, which corresponds to approximately 185 WT antibodies per virus. This result demonstrates that complement-mediated neutralization occurs at antibody-binding levels well below the maximum occupancy but higher than those required by TRIM21, which can neutralize infection 10-fold with as little as 5 antibodies per virus (McEwan et al., 2012).

Having established that C1q mediates Ad5 neutralization in the presence of the mAb 9C12-WT, we tested whether this also occurs with serum IgG. Since we have shown that a threshold of mAb 9C12 coating is required for efficient C1q-dependent neutralization, we tested whether this degree of antibody coating can be achieved by polyclonal serum. First, we determined that the concentration of Ad5 specific antibodies in NHS is 97.2 μg/mL (Figure S1F), which is significantly higher than the concentration of 9C12 required for efficient C1q-dependent neutralization. We then compared binding of polyclonal serum antibody and binding of monoclonal 9C12 to Ad5 at matched Ad5-specific antibody concentrations and found that polyclonal antibodies coated the virus as efficiently as 9C12 (Figure S1G). Next, we performed a time course and incubated virus with either NHS or C1q-depleted serum (Figure 1D). We observed increasing viral neutralization over time using NHS, but not C1q-depleted serum, an effect that was strictly dependent on the presence of IgG in the serum (Figure 1E).

Given that C1q is able to neutralize Ad5 infection in the presence of both mAb and polyclonal serum, we sought to determine whether other members of the complement cascade are required. We found that while 9C12 neutralized Ad5 infection in C2- and C3-depleted sera as efficiently as in complete NHS, it was unable to do so in C1q- and C4-depleted sera (Figure 1F), indicating that C4 is required in addition to C1q. To further confirm that the observed Ad5 neutralization was solely dependent on C1 and C4, we attempted to reconstitute activity using only the purified proteins (Figure 1G). Keeping the concentration of C1 constant, we performed a titration of C4 and observed dose-dependent neutralization of Ad5. The EC_50_ of C4-mediated neutralization was determined as 45.5 μg/mL (SEM ± 3.0), a concentration of C4 ∼10-fold lower than normal serum levels and therefore well within the physiological range. Importantly, neutralization depended on the presence of an intact C1 complex (Figure S2A) and interaction of C1q with antibody (Figure S2B), indicating the activation of the classical pathway.

### Activation of the Complement Cascade Results in Deposition of C4b on the Ad5 Capsid

**Figure 2:**
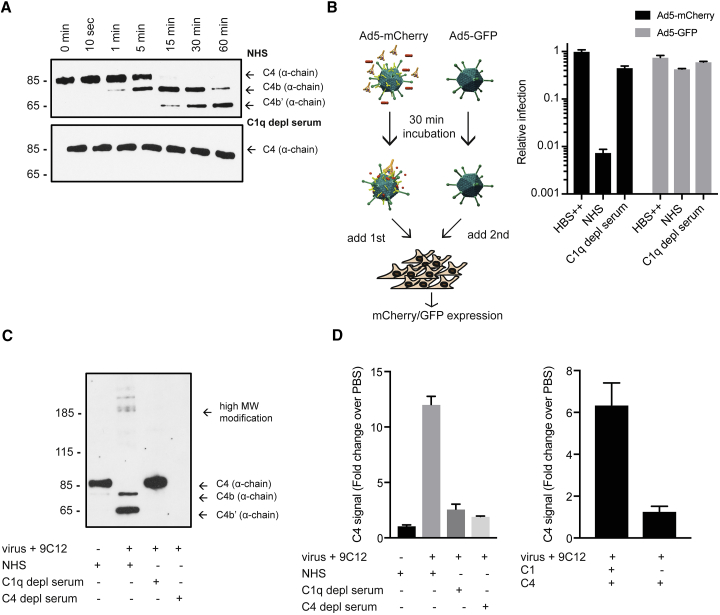
Activation of the Complement Cascade in Presence of Ad5 and 9C12 Results in Deposition of C4b on the Ad5 Capsid (A) Western blot of C4 (α-chain) cleavage in NHS (top) or C1q-depleted serum (bottom) in the presence of Ad5 and 9C12-WT over 60 min. See also Figure S2E. (B) Experimental set up (left): Ad5-mCherry+9C12-WT were incubated with HBS++/serum for 1 h at 37°C. Ad5-GFP was incubated with HBS++. Ad5-mCherry was added to HeLa TRIM21 KO cells immediately followed by Ad5-GFP. Right: relative infection of Ad5-mCherry and Ad5-GFP. x axis labeling corresponds to the buffer/serum that was incubated with Ad5-mCherry. (C) Western blot of C4 (α-chain) cleavage in NHS, C1q-depleted serum, or C4-depleted serum in the presence of Ad5 and 9C12-WT as indicated. (D) Elisa for C4 using serum (left) or purified protein (right). Ad5+9C12-WT were incubated with the indicated serum or purified protein and pelleted over a sucrose gradient. Error bars depict mean + SD of three replicates acquired in one representative experiment (A and D). Original western blots are included in Figure S6.

Classical pathway initiation relies on the interaction of C1q with antibody to activate the C1-associated proteases (Figure S2C), which, in the first step of the complement cascade, convert C4 into C4a and the highly reactive thioester C4b (Figure S2D). To directly test for complement activation, we incubated virus and antibody with NHS and monitored the generation of C4b over time (Figure 2A). We observed cleavage of C4 after only 1 min of incubation, and after 15 min, a large proportion of the C4 present in the serum was converted into C4b. This conversion was crucially dependent on an intact complement cascade, as we did not observe cleavage of C4 in C1q-depleted serum (Figure 2A).

Activation of the complement cascade and generation of the C4b and C4a fragments yields two hypotheses of how Ad5 neutralization could occur: either liberation of the C4a fragment could act on target cells and render them less permissive to Ad5 infection or generation of the highly reactive thioester C4b results in C4b deposition on the virus, impacting its ability to productively infect. To test which scenario is more likely, we made use of the fact that Ad5 can be engineered to express different transgenes (Figure 2B). Ad5-mCherry was incubated with antibody and NHS for 30 min to allow for complement activation and C4a generation as well as C4b deposition, while Ad5-GFP was kept under control conditions. Ad5-mCherry was added to cells, followed by Ad5-GFP. If neutralization were caused by an effect on the target cells, we would expect equal neutralization of both Ad5-mCherry and Ad5-GFP. However, if neutralization were caused by a direct modification of the viral capsid through C4b deposition, we would only expect neutralization of Ad5-mCherry. This latter outcome is what we observed; Ad5-mCherry was neutralized strongly in a C1q-dependent manner, while Ad5-GFP infection was unaffected (Figure 2B), indicating that neutralization requires direct modification of the viral capsid.

C4b contains a highly reactive thioester that is known to covalently attach to any nearby proteins. Consistent with this property, we observed C4-specific high-molecular-weight modifications upon activation of the complement cascade (Figure 2C). These started to appear after 15 min of incubation (Figure S2E) and likely represent C4b attachment to surrounding serum proteins. To test whether C4b also attaches to the Ad5 capsid, we incubated virus and 9C12-WT with NHS and pelleted the complex over a sucrose gradient to remove any proteins not associated with the viral capsid (Figure 2D). While no C4 was pelleted if NHS alone was loaded onto the gradient, we observed a strong C4 signal when virus and antibody were incubated with NHS, indicating that C4b was indeed deposited on the capsid. Again, this was dependent on an intact complement cascade since no C4 was pelleted if C1q- or C4-depleted serum was used. Similarly, we observed a strong signal for C4 if virus and antibody were incubated with C1 and C4, but not if they were incubated with C4 alone (Figure 2D). Taken together, these data suggest that C4b covalently attaches to the viral capsid and mediates neutralization of Ad5.

### C4b Deposition Does Not Prevent Ad5 Attachment or Internalization

**Figure 3:**
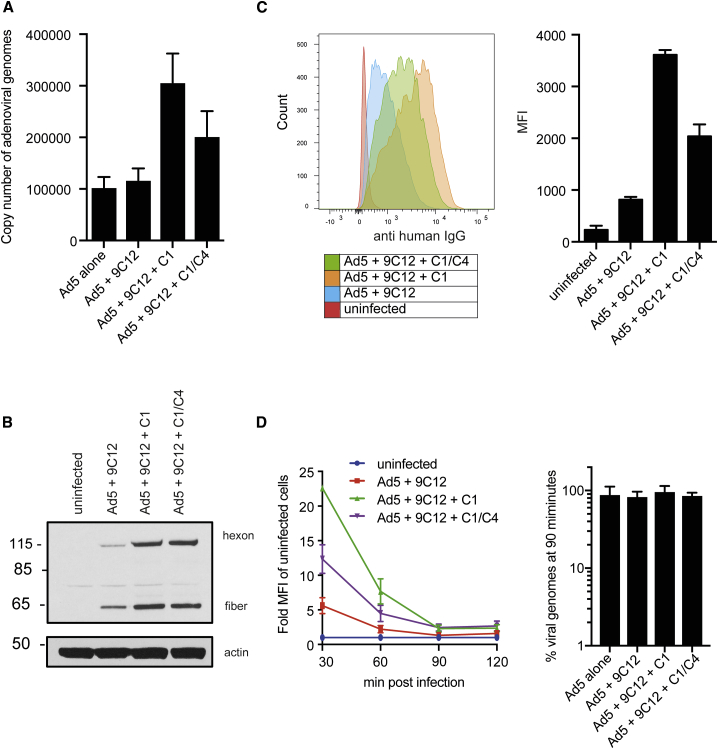
C1 and C4 Do Not Prevent Virus Attachment or Internalization Ad5 associated with HeLa TRIM21 KO cells after 30 min of continuous infection in the presence of C1 and C4 (A–C). (A) The viral copy number was determined by qPCR. (B) Western blot for adenovirus and actin. (C) Cells were stained with an anti-human IgG antibody and analyzed by flow cytometry. Histograms (left) and MFI (right) are depicted. (D) Internalization assay of virus after 30 min of continuous infection. Cells were harvested every 30 min, stained with an anti-human IgG antibody, and analyzed by flow cytometry (left). See also Figure S3C. qPCR of viral genomes present at 30 min and 90 min post infection (right). Viral genomes were normalized to the number of viral genomes present at 30 min post infection. Error bars depict the mean + SD of three replicates acquired in one representative experiment (A and C) or mean + SEM of nine replicates acquired in three independent experiments (D).

Next, we investigated the mechanism by which C4b deposition on the Ad5 capsid interferes with infection. We first tested whether C4b deposition could interfere with binding to host cell receptors and quantified the number of cell-associated viruses by qPCR (Figure 3A). Interestingly, we did not observe reduced attachment in the presence of C1 and C1/C4; instead, more viral copies were detected in the presence of complement. The same result was obtained when quantifying viral particles by western blot (Figure 3B) and flow cytometry (Figure 3C). Since a known neutralization mechanism by complement is the aggregation of viral particles (Oldstone et al., 1974), we used nanoparticle tracking analysis to demonstrate that C1q does not aid the formation of immune complexes (Figure S3A), which could have explained the increased attachment. Depletion of the main Ad5 receptor coxsackie-and-adenovirus receptor (CAR), however, revealed that in the presence of C1, virus attachment became less dependent on CAR (Figure S3B). This indicates that C1 may mediate binding to another surface receptor, which is consistent with the expression of C1q-receptor and binding proteins at the cell surface. An attractive candidate might be gC1qBP, whose expression is not restricted to phagocytes but has been shown to be expressed on the plasma membrane of various cell types (Soltys et al., 2000). Next, we hypothesized that complement might interfere with the internalization process of viral particles. To test this, we performed a time course to track viral entry. Cells were washed 30 min post Ad5 infection, harvested in 30 min intervals, and stained for surface-bound Ad5 particles (Figures 3D and S3C). While no surface-bound virions could be detected by flow cytometry 90 min post infection, all viral genomes that were associated with the cells at 30 min post infection were still associated with the cells at 90 min post infection (Figure 3D), indicating that the virions were internalized rather than shed into the medium.

### C4b Deposition Prevents Ad5 Capsid Disassembly, Endosomal Escape, and Entry into the Cytosol

**Figure 4:**
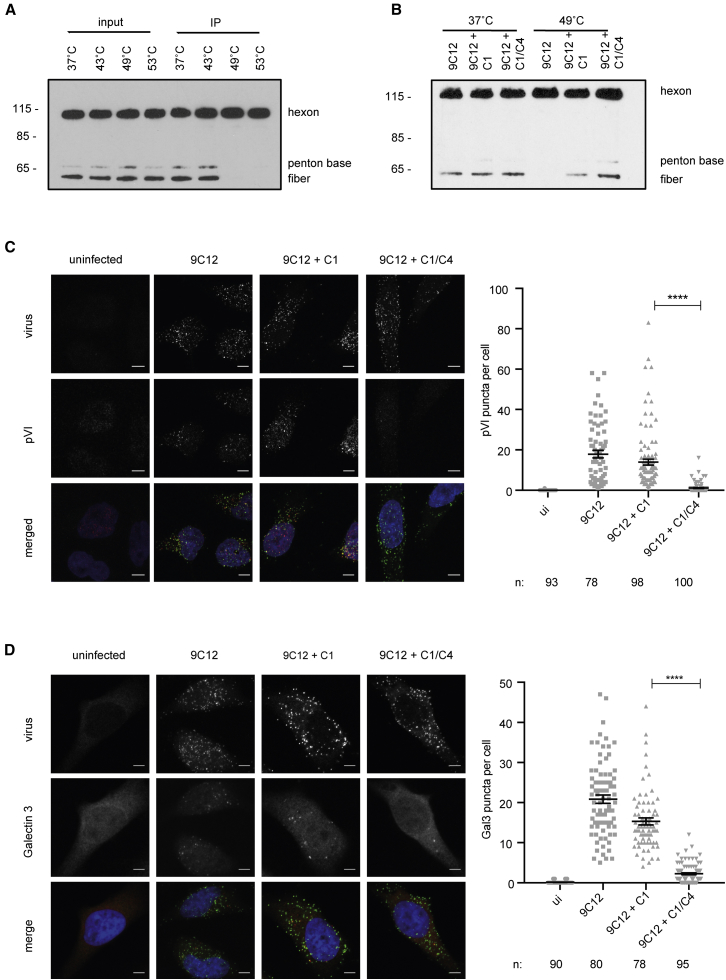
C1 and C4 Prevent Adenoviral Protein VI Exposure and Endosomal Escape (A) IP of Ad5 with 9C12 after Ad5 was incubated at the indicated temperatures for 30 min. WB: anti-adenovirus. (B) IP after Ad5 and 9C12 were complexed with C1 or C1/C4 and then incubated at 37°C or 49°C for 30 min. WB: anti-adenovirus. (C and D) HeLa TRIM21 KO cells were infected for 30 min in the presence of 9C12 or 9C12+ complement. Error bars depict the mean ± SEM of the indicated number of cells (n) acquired in three independent experiments. Scale bar, 5 μm. (C) Left: Ad5 staining is displayed in green; protein VI staining is depicted in red. Right: quantification of protein VI puncta per cell in the indicated conditions. (D) Left: Ad5 staining is displayed in green; Galectin-3 staining is depicted in red. Right: quantification of Galectin-3 puncta per cell in the indicated conditions. Original western blots are included in Figure S6.

Host-cell-receptor interaction is crucial for productive Ad5 infection. Ad5 engages two cell surface receptors to trigger entry: it interacts with CAR (Bergelson et al., 1997) through its trimeric fiber protein while engaging αvβ3/5 integrins through an RGD motif in its penton base (Bai et al., 1994, Wickham et al., 1993). While CAR undergoes a drifting motion on the plasma membrane, the integrin remains static, thereby promoting the release of fiber and penton base from the Ad5 capsid. This results in the release of the membrane-lytic protein VI, which is essential for endosomal lysis and entry of Ad5 into the cytosol (Luisoni et al., 2015, Burckhardt et al., 2011, Meier et al., 2002, Maier et al., 2012, Wiethoff et al., 2005). To test whether C4b deposition on the virus interferes with this capsid disassembly process and thus membrane lysis, we analyzed the three processes that need to occur: fiber shedding, protein VI exposure, and membrane lysis. To examine fiber shedding in the presence of deposited C4b, we exploited the fact that Ad5 sheds its fiber not only in response to mechanical cues but also in response to heat (Smith and Nemerow, 2008, Wiethoff et al., 2005). We incubated the Ad5 capsid at different temperatures and then performed immunoprecipitation (IP) with 9C12, which precipitates the intact capsid (Figure 4A). Upon heating Ad5 to 49°C, fiber and penton base were no longer detected by western blot, indicating that they were not associated with the capsid but had been shed. When the same experiment was repeated in the presence of C1 and C4 (Figure 4B), both fiber and penton base were still associated with the Ad5 capsid when it was heated to 49°C, indicating that C4b deposition on the viral capsid does interfere with Ad5 capsid disassembly.

Next, we investigated whether C4 prevents exposure of protein VI during infection by confocal microscopy (Figure 4C), which only detects partially disassembled capsids where the pVI epitope is unmasked (Burckhardt et al., 2011). We observed good protein VI exposure in the presence of 9C12-WT and C1, but the amount of protein VI staining significantly decreased in the presence of C1 and C4, demonstrating that C4b deposition inhibits protein VI exposure. This is consistent with the lack of capsid disassembly indicated by blotting for fiber and penton base. Finally, we tested whether the reduced exposure of membrane-lytic protein VI is sufficient to prevent the virus from penetrating the endosomal membrane. We used Galectin 3 as a marker for endosomal lysis since it is recruited to ruptured endosomes (Aits et al., 2015, Maier et al., 2012, Luisoni et al., 2016) and forms distinct puncta proportional to the multiplicity of infection (MOI) of Ad5 used (Figure S4). Similarly to the protein VI staining microscopy, we observed nice puncta formation in response to Ad5 infection in the presence of 9C12-WT and C1; however, significantly less Gal3 puncta were observed during infection in the presence of C1 and C4 (Figure 4D). This suggests that in the presence of complement, Ad5 penetrates endosomes less efficiently.

**Figure 5:**
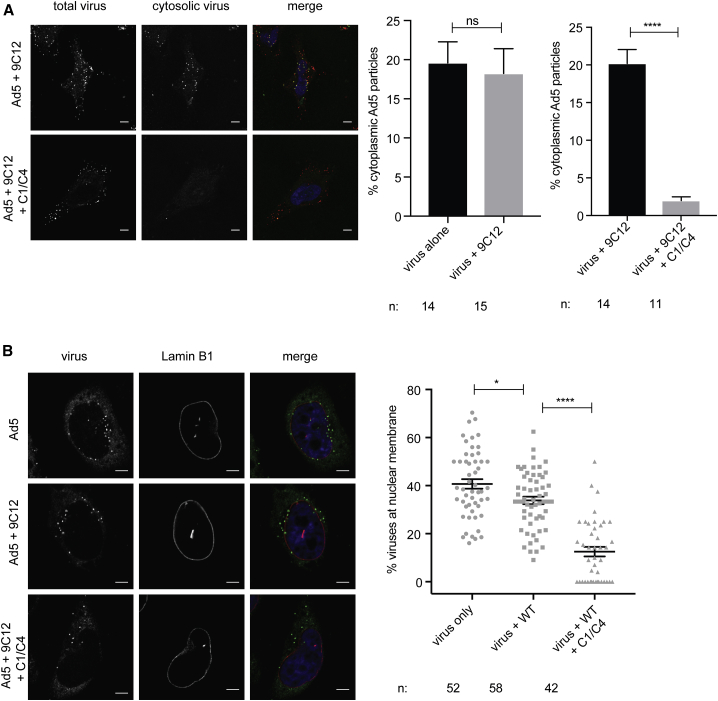
C1 and C4 Prevent Ad5 Entry into the Cytosol and Trafficking to the Nucleus (A) Cytosolic Ad5 staining is displayed in green, total Ad5 staining is displayed in red (left). Percentage of cytosolic Ad5 after infection with Ad5 alone or Ad5+9C12 (middle). Percentage of cytosolic Ad5 after infection with Ad5+9C12 or Ad5+9C12+C1/C4 (right). (B) Ad5 staining is displayed in green; lamin B1 staining is depicted in red (left). Quantification of viruses at the nuclear membrane (right). Scale bar, 5 μm. Error bars depict the mean ± SEM of the indicated number of cells (n) acquired in one representative experiment.

To directly demonstrate that viruses are trapped in endosomes in the presence of complement and prevented from entering the cytosol, we used a variation of the membrane penetration assay described by Suomalainen et al. (2013). We electroporated HeLa cells with an anti-Fab-FITC antibody and infected them with Ad5 in the presence of 9C12-WT and complement. Any viruses that have penetrated the endosome and escaped into the cytosol will be bound by the electroporated anti-Fab antibody. Cells were then fixed, permeabilized, and stained with an anti-Fab followed by an AF647-labeled secondary antibody. Therefore, all cytosolic viruses will acquire a dual FITC and AF647 signal, while endosomal viruses will only be labeled with the AF647 signal. Investigating whether 9C12 alone alters egress into the cytosol, we found that around 20% of viruses were cytosolic regardless of the presence of 9C12 (Figure 5A, middle). Upon the addition of complement, however, the percentage of cytosolic viruses dropped to less than 2% (Figure 5A, left and right), further corroborating our data that C1 and C4 specifically interfere with virus entry into the cytosol.

Once Ad5 has escaped the endosome, hexon recruits dynein and travels along microtubules to the nucleus, where it interacts with the nuclear pore complex to deliver the viral genome into the nucleus (Bremner et al., 2009, Leopold et al., 2000, Suomalainen et al., 1999, Cassany et al., 2015, Trotman et al., 2001). Given that complement inhibits viral entry into the cytosol, we hypothesized that this should result in fewer viruses reaching the nuclear membrane. To confirm this, we stained the nuclear membrane with Lamin B1 and scored the percentage of viruses associated with the nuclear membrane. Indeed, 2 h post infection, only 12.5% of viruses were associated with the nuclear envelope in the presence of complement, compared to 34.1% in the presence of 9C12 (Figure 5B). Interestingly, 9C12 also seemed to affect trafficking to the nucleus since there was a small decrease in the percentage of viruses at the nuclear membrane compared to Ad5 alone (40.3%) (Figure 5B). This is consistent with reports that anti-hexon antibodies can interfere with transport of virus to the nucleus (Bremner et al., 2009).

Taken together, these data suggest that deposition of C4b on the Ad5 capsid prevents efficient uncoating of the virus in response to environmental cues. This results in reduced protein VI exposure, preventing the virus from penetrating the endosome to gain access to the cytosol and travel to the nucleus.

### Complement and TRIM21 Mediate Blocking of Transgene Expression *In Vivo*

**Figure 6:**
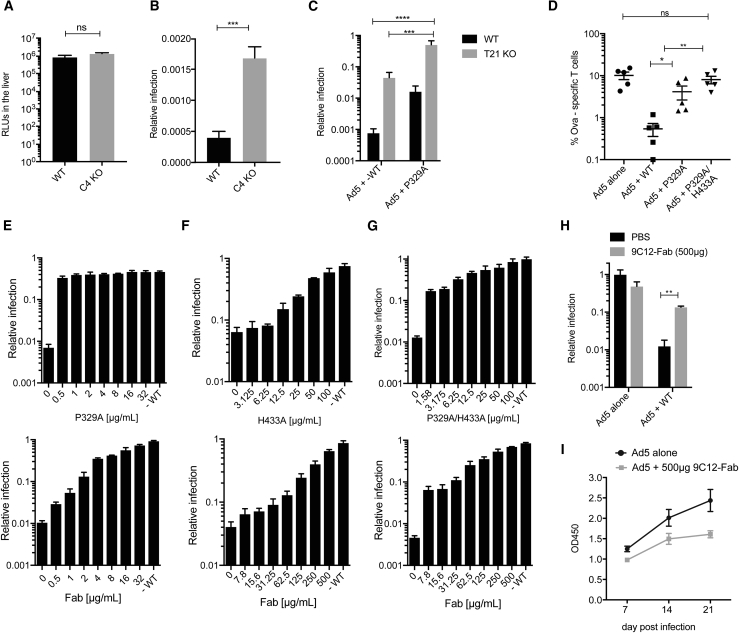
Complement and TRIM21 Mediate Block to Transgene Expression *In Vivo* (A) Relative light units (RLUs) indicating absolute levels of Ad5 infection in WT and C4 KO animals. (B) Relative infection of Ad5-Luc in WT and C4 KO mice using 9C12-WT. (C) Relative infection of Ad5-Luc in WT and T21 KO mice using 9C12-WT and mutant P329A. (D) SIINFEKL (SL8) specific CD8 T cell frequency in the blood of mice i.v. immunized with Ad5-Ova in the presence of 9C12-WT and mutants P329A and P329A/H433A. (E–G) Competition assays using 9C12 mutants (top) and m9C12 Fab (bottom). Neutralization assay using 1 μg/mL 9C12-WT in HeLa T21 KO cells titrating in P329A (E), in 293T-WT cells titrating in H433A (F), and in 293T-WT cells titrating in P329A/H433A (G). (H) Relative infection of Ad5-Luc in WT mice using 0.5 μg 9C12-WT in the presence of 500 μg m9C12 Fab. (I) Anti-Ad5 antibodies in mouse serum at the indicated days post infection. Groups consisted of 3–9 mice; error bars depict the mean ± SEM (A–D, H, and I). Error bars depict the mean + SD of three replicates acquired in one representative experiment (E–G).

Next, we investigated whether capsid inactivation by C1 and C4 is sufficient to inhibit viral infection *in vivo*. To this end, we assessed viral infectivity using an Ad5 vector carrying a luciferase transgene, which allows infection to be quantified by bioluminescence. Consistent with published data (Xu et al., 2013), we observed that knockout of C4 alone did not impact on transgene expression (Figure 6A). In contrast, co-infection in the presence of 9C12-WT substantially decreased transgene expression, consistent with potent neutralization, and this effect was reduced in C4 KOs (Figure 6B). Significant antibody-dependent neutralization remained in KOs; however, we have previously shown that the cytosolic antibody receptor TRIM21 mediates a potent antibody block to adenovirus *in vivo* (Bottermann et al., 2018). To separate the contributions of TRIM21 and complement to Ad5 neutralization, we compared 9C12-WT with the P329A mutant, which ablates C1q binding, and performed Ad5 infections in WT and TRIM21 KO mice (Figure 6C). The P329A mutant reduced neutralization in both WT and TRIM21 KO animals, confirming that this phenotype is TRIM21-independent and in agreement with our *in vitro* neutralization data (Figure 1A). Moreover, while a significant proportion of the >1,000-fold reduction in transgene expression was mediated by TRIM21, in accordance with our previous findings (Bottermann et al., 2018), transgene expression was not fully restored in the TRIM21 KO (Figure 6C). Importantly, removing both TRIM21 and complement (P329A in TRIM21 KOs) almost fully restored transgene expression to levels observed in the absence of antibody. These data support the idea that adenovirus neutralization does not rely on either complement or TRIM21 but that these two humoral immune factors work synergistically to prevent infection. Notably, this is also in line with our observation that C1q binding does not impact on TRIM21 binding to the antibody (Figure S1E) and, therefore, that these two immune factors can carry out their effector functions simultaneously.

As several viruses are known to antagonize complement, we looked for possible Ad5 antagonism of complement on the transcriptional level by mining an existing dataset that analyzes changes in gene expression after Ad5 infection in mouse liver (GEO: GSE119119). We used a gene set for the complement pathway (Kegg pathway map 04610) and found that *C1qa*, *C1qb*, and *C1qc* were among the most strongly downregulated transcripts after Ad5 infection (Figure S5A). Since C1q is predominately produced by macrophages (Fraser et al., 2015) and Ad5 infection rapidly depletes Kupffer cells in the liver (Manickan et al., 2006), this might contribute to reduced C1q transcription. Depleting Kupffer cells and other macrophages using clodronate liposomes (van Rooijen and Hendrikx, 2010) prior to Ad5 administration led to a reduction in *C1qa* levels, both upon Ad5 infection as well as 48 h after administration of clodronate liposomes (Figure S5B). This reduction in C1q expression by either specific downregulation or Kupffer cell depletion is potentially advantageous for the virus. In line with this observation, it has been shown that serum C1q levels are decreased in individuals with acute adenoviral conjunctivitis (Gupta and Sarin, 1984).

While a strong humoral immune response is beneficial to prevent infection, it is problematic during viral gene therapy or vaccination. Ad5 is the most widely used gene therapy vector, but its application is restricted by high seroprevalence within the human population. We therefore investigated how complement contributes to the pre-immune block to adenoviral-vector-based vaccination. We inoculated mice with an Ad5 vector expressing the model antigen ovalbumin (Ad5-Ova), which contains the immunodominant CD8 T cell epitope SIINFEKL (SL8), and anti-SL8 cytotoxic T lymphocytes (CTLs) can be quantified after immunization by using specific major histocompatibility complex (MHC) class I tetramers. We intravenously administered Ad5-Ova and analyzed the percentage of SL8-specific T cells 10 days post infection (Figure 6D). While we observed a strong inhibition of CD8 T cell induction in the presence of 9C12-WT, use of the P329A mutant significantly alleviated this block to T cell induction. Moreover, use of the double mutant P329A/H433A, which prevents both C1q and TRIM21 binding (Figure S1E), completely restored CD8 T cell induction (Figure 6D), further supporting the notion that complement and TRIM21 inhibit viral infection synergistically.

Given that complement and TRIM21 represent a significant block to adenoviral gene therapy, we sought a way to inhibit their effect. A potential solution is to interfere with antibody and therefore C1q and TRIM21 binding. We hypothesized that this could be accomplished by pre-coating adenovirus with antibody-binding mutants or Fab fragments that lack the Fc-binding region entirely. We incubated Ad5 with 1 μg/mL 9C12-WT and titrated in increasing amounts of mutant antibody variants or mouse-derived 9C12 Fab (m9C12 Fab) (Figures 6E–6G). We found that mutant antibodies and Fab were able to interfere with complement-mediated neutralization at low ratios (Figure 6E), while high excess was needed to abrogate TRIM21-dependent neutralization (Figure 6F). Determining the efficiency of pre-coating in the presence of both complement and TRIM21 neutralization mechanisms, we observed that a low concentration of antibody mutant or Fab was sufficient to recover about 10-fold of infection, likely reflecting the C1q-dependent neutralization, while higher concentrations were needed to dose-dependently restore infection to no antibody control levels, likely reflecting the TRIM21 component of neutralization (Figure 6G). These data are consistent with the higher antibody-coating requirement for complement-mediated neutralization (Figures 1A and 1C).

Having demonstrated that Fab pre-coating can rescue Ad5 infection *in vitro*, we investigated whether this strategy could also rescue transgene expression *in vivo*. When challenging mice with Ad5-luciferase in the presence of 1,000-fold excess m9C12-Fab, we were able to increase transgene expression 10-fold (Figure 6H). These data support a recent report that pre-coating with an artificial scFv trimer increases transgene delivery (Schmid et al., 2018). Finally, we tested whether a pre-coating strategy can inhibit the generation of a specific immune response in naive mice. We immunized mice either with Ad5 alone or Ad5 and m9C12-Fab and assessed the antibody response on days 7, 14, and 21 post infection (Figure 6I). Interestingly, we observed a reduced antibody response in the presence of Fab, indicating that co-administration of the vector with m9C12-Fab might be a powerful tool not only to circumvent pre-existing immunity but also to prevent a strong antibody response if the vector is administered to a naïve individual and might thus facilitate re-administration.

## Discussion

Here, we show that complement C4 inhibits viral infection both *in vitro* and *in vivo*. Using the model non-enveloped virus Ad5, we demonstrate that C4 is recruited to viral capsids via the classical complement pathway, which results in C4 cleavage. Covalent coupling of C4b to the viral capsid interferes with the carefully orchestrated stepwise uncoating process that is essential for adenovirus infection (Greber et al., 1993). Importantly, these data reveal that C4 possesses intrinsic neutralization activity and an immune function that is independent of all downstream complement components.

The C4 antiviral mechanism we uncovered is analogous to Ad5 neutralization by human α-defensins, which have been shown to stabilize the Ad5 vertex region by bridging fiber and penton base and thereby prevent fiber shedding, protein VI exposure, and endosomal escape (Smith and Nemerow, 2008, Flatt et al., 2013). At low concentration, α-defensins preferentially bind the vertex region, while C4b non-specifically attaches to hydroxyl and amino groups close to its site of activation, rendering the idea of C4b coupling being more efficient in the vertex region unlikely. This implies that a threshold coating of the Ad5 capsid by C4b is required, consistent with the higher antibody concentration requirement for complement-dependent neutralization (Figure 1A) and the low concentration requirement for Fab-blocking (Figure 6E). A higher antibody concentration may be important to activate C1q as well as to ensure good coverage of capsid by C4b. In this context, it is also possible that the pseudo-hexagonal base of the trimeric hexon protein (Liu et al., 2010) promotes IgG hexamer formation on the virus surface, which has been shown to facilitate C1q binding (Diebolder et al., 2014), thus making Ad5 a particularly good target for complement-mediated neutralization.

Importantly, at low antibody concentrations, where C4 deposition is inefficient, neutralization by the cytosolic antibody receptor TRIM21 is still effective. Hence, these two mechanisms can be seen as operating synergistically: at higher antibody concentrations, neutralization is largely C4 dependent and at lower concentrations, TRIM21 dependent. Viruses can be inactivated by C4, in which case they never make it to the cytosol or they escape their endosomes only to be detected by TRIM21 and targeted for proteasomal degradation.

Consistent with complement and TRIM21 being antibody-dependent neutralization mechanisms, patients with agammaglobulinemia are more susceptible to adenovirus infections, which can be severe or even fatal (Schultz et al., 2008). Since there are no known cases of fatal adenovirus infections in complement-deficient patients, it would be interesting to investigate whether TRIM21 is important in combating adenovirus infection in the immunocompetent host; however, as of yet, there are no case studies of patients with known TRIM21 deficiency.

Previously described complement-mediated virus neutralization mechanisms generally fall under three categories, neutralization by aggregation, neutralization by lysis, or neutralization by complement protein deposition (Cooper and Nemerow, 1983). This last type of neutralization has been shown for several viruses, such as vesicular stomatitis virus, vaccinia virus (Leddy et al., 1977), and Newcastle disease virus (Linscott and Levinson, 1969); however, no detailed mechanism has been demonstrated. The C1/C4-mediated neutralization we have described represents a comprehensive complement-mediated viral neutralization mechanism. In support of this mechanism, Xu et al. (2013) provided evidence for C1/C4-mediated neutralization *in vivo* showing that Factor X shields Ad5 from natural IgM binding which becomes redundant in C1q and C4 KO mice. Factor X binding could therefore be considered a viral escape mechanism that specifically evolved to avoid C4 neutralization. Such a hypothesis is supported by the fact that Factor X is still needed in C3 KOs and that C4 neutralization is very sensitive to blocking Fabs, which prevent antibody binding similar to Factor X. While Factor X blocks natural IgM, it is very likely insufficient to prevent binding by the specific anti-Ad5 mAb 9C12, given the severe inhibition of viral transgene expression we observed *in vivo* upon antibody passive transfer. This may reflect the distinct binding sites of this mAb (Bottermann et al., 2016) and Factor X (Alba et al., 2011) or that the former outcompetes the serum protein.

However, 9C12 binding can be prevented using Fab fragments, which rescue transgene expression *in vivo*. Therapeutically, this could be combined with the use of scFvs or DARPins that target Ad5 to the desired site within the body (Schmid et al., 2018). Moreover, we also found that incubating Ad5 with Fab fragments prior to the initial injection resulted in a reduced Ad5-specific antibody titer. This might be a result of epitope masking as described in antibody-mediated immune suppression (Getahun and Heyman, 2009). We suggest that a strategy of pre-coating might be particularly beneficial when multiple administrations of adenovirus-based vectors are required.

## STAR★Methods

### Key Resources Table

**Table:** 

REAGENT or RESOURCE	SOURCE	IDENTIFIER
**Antibodies**		

Rabbit polyclonal anti adenovirus protein VI (affinity purified)	Urs F. Greber (Burckhardt et al., 2011)	N/A
Goat anti-Rabbit IgG (H+L) AF647	Thermo Fisher Scientific	Cat# A-21245; RRID: AB_2535813; Dil: 1:500
Galectin 3 Monoclonal Antibody (M3/38) PE	Thermo Fisher Scientific/ eBioscience	Cat# BMS1043; RRID: AB_10597892; 1μg/mL
Anti-Human IgG (Fab specific)-FITC antibody produced in goat	Sigma-Aldrich	Cat# F5512; RRID: AB_259649; Dil: 1:200
Anti-Human IgG (Fab specific)- antibody produced in goat	Sigma-Aldrich	Cat# I5260; RRID: AB_260206; Dil: 1:200
Donkey Anti-Goat IgG (H+L) Antibody, AF647	Thermo Fisher Scientific	Cat# A-21447; RRID: AB_141844; Dil: 1:1000
Lamin B1 antibody	Abcam	Cat# ab16048; RRID: AB_10107828; Dil: 1:1000
Goat Anti-Rabbit IgG (H+L) Antibody AF568	Thermo Fisher Scientific	Cat# A-11011; RRID: AB_143157; Dil: 1:1000
Anti-Adenovirus antibody	Millipore	Cat# AB1056; RRID: AB_90213; Dil: 1:1000
52 kDa Ro/SSA (D-12) antibody (TRIM21)	Santa Cruz Biotechnology	Cat# sc-25351; RRID: AB_628286
Anti-C4b antibody	Abcam	ab181241; Dil: 1:1000
β-Actin Antibody (C4)	Santa Cruz Biotechnology	Cat# sc-47778 HRP; RRID: AB_2714189; 0.04μg/mL
Rabbit anti goat IgG (H/L) antibody	Bio-Rad / AbD Serotec	Cat# 5160-2104; RRID: AB_619999; Dil: 1:5000
Anti-Mouse IgG (whole molecule)-Peroxidase antibody produced in rabbit	Sigma-Aldrich	Cat# A9044; RRID: AB_258431; Dil: 1:5000
Anti-Rabbit IgG (whole molecule)-Peroxidase antibody produced in goat	Sigma-Aldrich	Cat# A0545; RRID: AB_257896; Dil: 1:5000
6X His tag antibody [HIS-1] (Alkaline Phosphatase)	Abcam	Cat# ab49746; RRID: AB_867457; Dil:1:5000
C1q complement antibody	Dako	Cat# A0136; RRID: AB_2335698; Dil:1:10000
Rabbit IgG HRP Linked Whole Ab antibody	GE Healthcare	Cat# GENA934; RRID: AB_2722659; Dil:1:10000
Anti human IgG (Fc-specifc) AP linked antibody	Sigma-Aldrich	Cat#:A9544; RRID: AB_258459; Dil: 1:5000
iTAg Tetramer/PE - H-2 Kb OVA (SIINFEKL)	MBL	Cat# TB-5001-1; 2μL/sample

**Bacterial and Virus Strains**		

Adenovirus 5 (E1/E3 deleted)	ViraQuest	VQAd CMV eGFP/mCherry

**Chemicals, Peptides, and Recombinant Proteins**		

Complement serum	CompTech	Cat#NHS
C1q depleted serum	CompTech	Cat#A300
C2 depleted serum	CompTech	Cat#A312
C3 depleted serum	CompTech	Cat#A314
C4 depleted serum	CompTech	Cat#A308
C1q	CompTech	Cat#A099
C1	CompTech	Cat#A098
C4	CompTech	Cat#A105
Adenovirus Type 5 Hexon	BioRad	MPP002

**Critical Commercial Assays**		

Human Complement C4 ELISA Kit	abcam	Cat#ab108825
RNeasy Micro Kit	Qiagen	Cat#74004
DNeasy Blood & Tissue Kit	Qiagen	Cat# 69504

**Deposited Data**		

Raw and analyzed data	(Bottermann et al., 2018)	GEO: GSE119119

**Experimental Models: Cell Lines**		

HEK293T	ATCC	Cat# CRL-3216; RRID: CVCL_0063
HeLa	ATCC	Cat# CCL-2; RRID: CVCL_0030
THP-1	ATCC	Cat#:TIB-202; RRID: CVCL_0006

**Experimental Models: Organisms/Strains**		

C57BL/6J	The Jackson Laboratory	JAX:000664; RRID: IMSR_JAX:000664
C57BL/6-*Trim21*^*tm1Hm*^/J	(Yoshimi et al., 2010)	JAX:010724; RRID: IMSR_JAX:010724
B6.129S4-*C4*^*tm1Crr*^/J	Provided by Michael C. Carroll	JAX:003643; RRID: IMSR_JAX:003643

**Oligonucleotides**		

TRIM21 targeting gRNA; 5’ATGCTCACAGGCT CCACGAA3’	(McEwan et al., 2017)	N/A
Ad5 Taqman probe; Ad5 for: TTGCGTCGGTG TTGGAGA, Ad5 rev: AGGCCAAGATCGTGAA GAACC, Ad5 probe: FAM-CTGCACCACAT TTCGGCCCCAC-TAMRA	(Bottermann et al., 2018)	N/A
CAR siRNA	Santa Cruz	sc-29906
Control siRNA	Santa Cruz	sc-37007

**Recombinant DNA**		

pSpCas9(BB)-2A-GFP (PX458)	(Ran et al., 2013)	Addgene #48138

**Software and Algorithms**		

FlowJo, LLC	Tree Star	https://www.flowjo.com/solutions/flowjo/downloads
GraphPad Prism version 7.00	GraphPad Software	https://www.graphpad.com/scientific-software/prism/
Fiji	(Schindelin et al., 2012)	https://fiji.sc/#download
ImageJ	(Schneider et al., 2012)	https://imagej.nih.gov/ij/download.html
Image J plugin ComDet v0.3.7	Eugene Katrukha	https://imagej.net/Spots_colocalization_(ComDet)

### Contact for Reagent and Resource Sharing

Further information and requests for resources and reagents should be directed to and will be fulfilled by the Lead Contact, Leo C. James (lcj@mrc-lmb.cam.ac.uk).

### Experimental Model and Subject Details

#### *In Vivo* Animal Studies

Husbandry and housing conditions of experimental animals confirms to standards set out under the UK Animals (Scientific Procedures) Act 1986 and the Medical Research Council Animal Welfare and Ethical Review Body. The same bodies approved the work and experimental protocols, together with human cell studies. Strains C57BL/6 (JAX:000664) and C57BL/6-*Trim21*^*tm1Hm*^/J (JAX:010724) were obtained from Jackson Laboratories. Strain B6.129S4-*C4*^*tm1Crr*^/J (JAX:003643) was a kind gift from Michael C. Carroll (Boston’s Children Hospital, Boston, USA). 6-8 week-old males and females (usually 20g – 30g) were used in infection experiments, which were conducted in accordance with the 19.b.7 moderate severity limit protocol and Home Office Animals (ScientificProcedures) Act (1986). No weight-matching or sex-matching was performed. Throughout the protocol, animals were weighed and observed daily for clinical signs of infection, which included subdued behavior, pilo-erection, hunched posture, ataxia, and paresis. Animals that reached the end of the experiment, lost more than 20% of initial body weight, or showed clinical signs that failed to improve over a 6h period were killed. Experimental groups consisted of 3-9 animals.

#### Cell Lines and Viruses

HEK 293T (RRID: CVCL_0063, female) and HeLa (RRID: CVCL_0030, female) cells were maintained in Dulbecco’s modified Eagle’s medium (Gibco, GlutaMAX) and THP-1 cells (RRID: CVCL_0006, male) were maintained in Roswell Park Memorial Institute medium (Gibco, GlutaMAX). Medium was supplemented with 100 U/ml penicillin, 100 μg/ml streptomycin, and 10% fetal calf serum (Gibco) and cells were grown at 37°C in a 5% CO_2_ atmosphere. Human Ad5 (E1/E3 deleted) was purchased from ViraQuest.

### Method Details

#### Human Serum and Complement Proteins

Human serum and human complement proteins were purchased from Complement Tech and all dilutions were made in HEPES-Buffered Saline (HBS++: 25mM HEPES (pH 7.4), 145mM NaCl, 0.5mM MgCl_2_, 0.15mM CaCl_2_).

#### Recombinant 9C12 Variants and m9C12 Fab Fragment

Vectors encoding 9C12-WT, P329A, LALA and H433A and m9C12 Fab have been described (Bottermann et al., 2016, Foss et al., 2016). The vector encoding 9C12-P329A/H433A was generated by site-directed mutagenesis using the vector encoding H433A as template (Genscript). The antibodies were produced in HEK293E cells by transient transfection using Lipofectamine 2000 (ThermoFisher) and purified using a CaptureSelect C_H_1 specific column (ThermoFisher) followed by size exclusion chromatography on a Superdex 200 10/300 column (GE Healthcare).

#### Neutralization Assays

293T or HeLa cells were plated at 5x10^4^ cells per well in a 24-well plate and allowed to adhere over night.

##### TRIM21-Mediated Neutralization

Ad5 (ViraQuest) was diluted to 1x10^9^ pts/mL and mixed 1:1 with the indicated antibody concentration and allowed to complex for 30 min at room temperature. 12 μL of the virus:antibody complex was added to cells in 500 μL cDMEM or serum-free medium.

##### Complement-Mediated Neutralization with 9C12

Ad5 (ViraQuest) was diluted to 5x10^9^ pts/mL and mixed 1:1 with the indicated antibody (15 μg/mL) and allowed to complex for 30 min at room temperature. Human serum was added to a final dilution of 1:30 or 1:60 (if applicable, C1q was added to the serum at 200μg/mL). For C1/C4 mediated neutralization C1 was added to a final concentration of 0.2 - 50 μg/mL^∗^, incubated for 30min at RT, followed by addition of C4 to a final concentration of and 130 μg/mL, unless otherwise indicated. Reactions were made up of equal volumes of virus, antibody and serum/complement proteins. The complexes were incubated at 37°C for 15min-60min (shorter incubation times for neutralization using serum, longer incubation time for neutralization using purified proteins) and 18 μL was added to each well in 500 μL serum-free medium. ^∗^*Due to batch variation, the C1 concentration used had to be determined batch-specifically*.

##### Complement-Mediated Neutralization with Serum IgG

Ad5 (ViraQuest) was diluted to 2x10^9^ pts/mL and mixed 1:1 with the indicated serum, diluted 1:10. C1q depleted serum was reconstituted with 200 μg/mL C1q protein as indicated. For IgG depletion, 10 μL NHS was incubated with 95 μL Protein G magnetic beads (Thermo Fisher) and immunoprecipitated for 5h at 4°C. Complexes were incubated for indicated time at 37°C and 12 μL was added to each well in 500 μL serum-free medium.

##### Competition Assays

The mutant antibodies or Fab fragments were mixed with WT antibody at the indicated ratio or concentration and neutralization assays were performed as described above.

Cells were harvested 20h post infection and the percentage of GFP or mCherry positive cells was determined by flow cytometry (*see* flow cytometry).

#### Protein VI Exposure/Galectin 3 Puncta Formation

5x10^4^ HeLa TRIM21 KO cells were plated on cover slips (Corning BioCoat, Poly-D-Lysine, 12 mm) in 24-well plates and allowed to adhere over night. Cells were infected in 150 μL SFM as described in Complement-mediated neutralization with 9C12 using 4x10^11^ pts/mL Ad5 and 20 μg/mL 9C12-WT. 30 min post infection cells were fixed and analyzed by immunofluorescence (*see* immunofluorescence). Z-stacks with 1 μm thick optical slices were acquired and protein VI and Galectin 3 puncta were quantified using NIS Elements 4.30 (Nikon). Images were acquired with a Zeiss LSM780 confocal microscope equipped with a 63x C-Apochromat 1.2 NA oil-immersion objective. Protein VI antibody and detailed protocols were obtained from Urs F. Greber and Maarit Suomalainen.

#### Quantification of Cytoplasmic versus Total Virus

HeLa TRIM21 KO cells were electroporated using the Neon Transfection System (Thermo Fisher). Cells were washed with PBS and resuspended in Buffer R (Thermo Fisher) at a concentration of 8x10^7^ cells/ml. For each electroporation reaction 8x10^5^ cells (10 μl) were mixed with 2 μl (total of 1μg) of goat anti-hIgG (Fab-specific)-FITC-conjugated antibody (Sigma; F5512), for the detection of Ad5 in the presence of 9C12, or 2 μL total of 9C12-WT (1 μg total) and goat anti-hIgG (Fab-specific)-FITC-conjugated antibody (Sigma; F5512, 1μg total), to detect Ad5 only. The mixture was taken up into a 10 μl Neon Pipette Tip (ThermoFisher) and electroporated using the following settings: 1400V, 20ms, 2pulses. Electroporated cells were transferred to medium supplemented with 10% fetal calf serum (Gibco) without antibiotics. 5x10^4^ HeLa TRIM21 KO cells were plated on cover slips (Corning BioCoat, Poly-D-Lysine, 12 mm) in 24-well plates and allowed to adhere over night. Cells were infected in 150 μL SFM as described in Complement-mediated neutralization with 9C12 using 4x10^11^ pts/mL Ad5 and 20 μg/mL 9C12-WT. Those viruses that escaped into the cytosol were bound by the electroporated antibody and appear as FITC-labelled particles. Total virus was visualised by permeabilizing cells and immunostaining with goat anti-hIgG (Fab-specific) (Sigma Aldrich; I5260) followed by anti-goat-AF647 (ThermoFisher; A21447). Thus, total virus appears as AF647-labelled particles and cytoplasmic virus appears as FITC+AF647 double-labelled particles. Quantification of colocalization between cytoplasmic and total virus was performed using the Image J plugin ComDet v0.3.7. In brief, particles were detected from single focal plane confocal images (60x60 μm; 1024x1024 pixels) in both FITC and AF647 channels independently with an approximate size of 5 (FITC) and 4 (AF647) pixels and intensity threshold of 4 (FITC) and 5 (AF647). Colocalization was determined based on maximum distance of 2 pixels between particles. >95% of FITC particles colocalized with AF647 particles, confirming that the FITC particles detected were indeed viruses.

Images were acquired with a Leica SP8 confocal microscope equipped with 63x PL APO 1.4 NA oil-immersion objective.

#### Quantification of Nuclear Envelope-Localized Virus

5x10^4^ HeLa TRIM21 KO cells were plated on cover slips (Corning BioCoat, Poly-D-Lysine, 12 mm) in 24-well plates and allowed to adhere over night. Cells were infected in 150 μL SFM as described in Complement-mediated neutralization with 9C12 using 4x10^11^ pts/mL Ad5 and 20 μg/mL 9C12-WT. 2h post infection cells were fixed and and immunostained with anti-hexon (9C12) and anti-Lamin B1 (ab16048) antibodies to detect viruses and the nuclear envelope respectively. To quantify nuclear envelope-associated viruses, the nuclear envelope region was segmented in Image J using an intensity threshold of 10 in the Lamin B1 channel. Virus particles were detected in both segmented and unsegmented images using the Image J plugin ComDet v0.3.7 (particle size, 4 pixels; intensity threshold, 5). Nuclear envelope-associated viruses were quantified by calculating the percentage of total virus particles present in the nuclear-envelope-segmented region.

Images were acquired with a Zeiss LSM780 confocal microscope equipped with a 63x C-Apochromat 1.2 NA oil-immersion objective.

#### Quantification of Viral Genomes

5x10^4^ HeLa cells were plated in 24-well plates and allowed to adhere over night. Cells were infected in 150 μL SFM as described in Complement-mediated neutralization with 9C12. 30 min post infection, cells were harvested by scraping and the RNA was extracted using an RNeasy Mini Kit (Quiagen). The viral copy number was determined by qPCR (*see* qPCR).

#### Quantification of Surface Bound and Internalized Viruses

5x10^4^ HeLa cells were plated in 24-well plates and allowed to adhere over night. Cells were infected in 150μL SFM as described in Complement-mediated neutralization with 9C12 using 4x10^11^ pts/mL Ad5 and 20 μg/mL 9C12-WT. Cells were harvested by scraping 30 min post infection (unless otherwise indicated) and analyzed by western blot (surface bound and internalized) and flow cytometry (surface bound only).

#### Fiber Shedding

Ad5 was diluted to 4x10^11^ pts/mL, mixed 1:1 with 9C12-WT (20 μg/mL) and allowed to complex for 30 min at RT. C1 and C4 were added to a final concentration of 30 μg/mL and 130 μg/mL, respectively. The complexes were allowed to form at 37°C for 60 min followed by incubation at the indicated temperatures for 20 min and diluted to 300 μL. 40 μL Protein G magnetic beads (Thermo Fisher) were added and immunoprecipitation was performed for 2h, at 4°C. Reactions were washed 3x with RIPA buffer (Sigma Aldrich) and fiber shedding was analyzed by western blot.

#### Serum C4 Cleavage

Ad5 was diluted to 1x10^11^ pts/mL, mixed 1:1 with 9C12-WT (20 μg/mL) and allowed to complex for 30 min at RT. Serum was added to a final concentration of 1:60 and complexes were incubated for the indicated amount of time at 37°C. Reactions were stopped by the addition of 4x LDS and 10% dTT. C4 cleavage was analyzed by western blot.

#### Dual Virus Infection

5x10^4^ HeLa cells were plated in 24-well plates and allowed to adhere over night. Ad5-mCherry was diluted to 5x10^9^ pts/mL, mixed 1:1 with 9C12-WT (3 μg/mL) and allowed to complex for 30 min at RT. Serum was added to a final concentration of 1:60 and complexes were incubated for 60 min at 37°C. Ad5-GFP (5x10^9^ pts/mL) was incubated with HBS++ only. 18 μL Ad5-mCherry was added to each well immediately followed by 18 μL Ad5-GFP. Cells were harvested 20h post infection and GFP and mCherry expression was assessed by flow cytometry (*see* flow cytometry).

#### C4b Deposition on the Viral Capsid

Ad5 was diluted to 1x10^11^ pts/mL, mixed 1:1 with 9C12-WT (20 μg/mL) and allowed to complex for 30 min at RT. Serum was added to a final concentration of 1:30 and C1 and C4 were added to a final concentration of 30 μg/mL and 130 μg/mL, respectively. The complexes were incubated at 37°C for 60 min before layered on top of 500 μL 30% sucrose and gradients were centrifuged for 1h, 4°C, at 45000 rpm (TLA-45/55, Beckman Coulter). The pellet was resuspended in 500 μL PBS and centrifuged for 90 min, 4°C, 21000 rcf. The pellet was resuspended in PBS and the C4 signal was assessed by Elisa (Abcam, ab108825) according to manufacturer’s instructions.

#### Generation of Knockout Cell Lines Using the CRISPR/Cas9 System

HEK293T and HeLa cells were seeded in 6-well plates (2.5 × 10^5^ cell/well) and allowed to adhere over night. Cells were transfected with 1 μg pX458 (Addgene, #48138) (Ran et al., 2013) encoding a TRIM21 targeting gRNA (5’ATGCTCACAGGCTCCACGAA3’). 24 hours post transfection cells were trypsinized and GFP positive cells were sorted into 96-well plates (1 cell/well) using FACS. 14 days after sorting HEK293T clones were assessed for an indel event by Western Blot and Sanger Sequencing.

#### siRNA Knockdown

siRNA mix was assembled in a well of a 6-well plate (Corning): 300 μL Opti-MEM (Thermo Fisher Scientific), 3 μL siRNA (Santa Cruz; siCAR: sc-29906, siControl: sc-37007) and 9 μL Lipofectamine RNAiMAX (Thermo Fisher Scientific). The mix was incubated for 15min and 2.5x10^5^ cells were added in DMEM (Gibco) + 10% FCS (Gibco).

#### Nanoparticle Tracking Analysis

Ad5 (ViraQuest) was diluted to 5x10^9^ pts/mL, mixed 1:1 with 9C12-WT (15 μg/mL) and allowed to complex for 30 min at room temperature. C1 and C4 were added to a final concentration of 30 μg/mL and 130 μg/mL, respectively. The complexes were incubated at 37°C for 15min-30min, diluted 5-fold and were analyzed using a NanoSight LM10 (Malvern Pananalytical).

#### Experimental Infections

Mice were injected with 0.5 μg–2.5 μg 9C12 in 100 μL endotoxin free PBS (Teknova) by lateral tail vein injection. 4h–24h later mice were injected with the indicated dose of Ad5 in 100 μL endotoxin free PBS (Teknova) by lateral tail vein injection. For macrophage depletion, mice were injected i.v. with 100μL clodronate liposomes (Liposoma) and infected with Ad5 48h after liposome injection.

#### *Ex Vivo* Viral Assays

##### Viral Gene Delivery Assay

Mice were given 0.5 μg to 2.5 μg 9C12 by i.v. administration. 4h later mice were given 2x10^10^ viral particles of Ad5-luciferase (ViraQuest) by i.v. administration. When indicated Ad5 was pre-incubated with 500 μg m9C12-Fab fragments. 48h post infection mice were culled by cervical dislocation and tissues were collected. Liver and spleen were homogenized in passive lysis buffer (25mM Tris-phosphate (pH 7.6), 2mM EDTA, 10% Glycerol, 1% Triton X-100) using TissueRuptor (Qiagen). The homogenates were cleared by centrifugation (5 min, 300xg), diluted 1:10, mixed 1:1 with luciferase substrate (Promega) and analyzed using a BMG PHERAstar FS plate reader. Relative infection was calculated from the luciferase signal from indicated tissues (RLUs virus+antibody/ RLUs virus only).

##### T Cell Assays

For T cell assays, mice were given 1 μg 9C12 by i.v. adminstration. 4h later mice were immunized with 1x10^8^ viral particles of Ad5-Ova (ViraQuest) by i.v. On day 10 animals were culled by CO_2_ asphyxiation and blood was harvested by cardiac puncture into heparinized tubes (Sarstedt). Frequency of the T cells specific for the immunodominant peptide SIINFEKL (Ova_257-264_) was analyzed by flow cytometry (*see* flow cytometry).

##### Antibody Titer

1x10^10^ viral particles of Ad5 were incubated with 500 μg m9C12-Fab fragments, or PBS control for 30 min at RT. Mice were infected with virus +/- Fab by lateral tail vein injection. Blood samples were collected via the tail vein at 7, 14 and 21 days post infection. Serum samples obtained via centrifugation of clotted blood samples were used to determine the presence of anti-adenovirus specific antibodies by Elisa. Briefly, 96-well polystyrene microtiter plates (Microlon, Greiner) were coated overnight at 4°C with Ad5 (5x10^9^ pts/mL) in PBS. Plates were washed three times with 0.05% Tween 20 in phosphate buffered saline (PBS-T) before blocking in 5% skimmed milk-PBS- T for 1 h at 37°C and then three PBS-T washes. Plates were then incubated for 1h at 37°C with 1:200 dilution of each serum sample in duplicate in 5% skimmed milk-PBS-T. After three washes with PBS-T, 50 μl of horseradish peroxidase (HRP)-conjugated anti-mouse IgG antibody (Sigma Aldrich) diluted 1:1000 in 5% milk PBS–T, was added to each well and incubated at 37°C for 1h. The plates were washed four times with PBS-T and bound antibody detected with 50μl tetramethylbenidine (TMB, Invitrogen) followed by incubation at room temperature for 10 min. The reaction was stopped with 1 M H_2_SO_4_ and the optical density (OD) was read at 450 nm using a BMG PHERAstar FS plate reader.

#### Western Blot

Cell pellets were lysed in RIPA buffer (Sigma-Aldrich), mixed with 4x NuPAGE LDS Sample Buffer (Thermo Fisher Scientific) + 10% dTT and incubated at 95°C for 15 min. If necessary, samples were sonicated prior to adding the LDS sample buffer. *In vitro* reactions were mixed with 4x NuPAGE LDS Sample Buffer (Thermo Fisher Scientific) + 10% dTT and incubated at 95°C for 15 min. Samples were loaded onto a SDS-PAGE Gel (Invitrogen) and run in 1x MOPS SDS Running Buffer (Invitrogen). The protein was transferred onto a nitrocellulose membrane using the iBlot1 system (Invitrogen) which was then blocked in 5% milk (Marvel) diluted in PBS+0.01% Tween (PBST). The primary and secondary antibodies were also diluted in 5% milk in PBST and the membrane was washed three times with PBST after antibody incubations. HRP-coupled secondary antibody was detected using ECL Prime Western Blotting Detection Reagent (GE Healthcare).

##### 1˚ Antibodies

anti-adenovirus (Millipore, AB1056): 1:1000 (detection from cellular extracts) and 1:10000 (detection from in vitro reaction), incubation performed ON at 4°C; anti-human-TRIM21 (Santa Cruz, D12): 0.4μg/mL, anti C4 (Abcam, ab108825): 1:1000, anti-human- β-actin-HRP (Santa Cruz, C4): 0.04 μg/mL

##### 2˚ Antibodies

anti-goat-HRP (BioRad): 1:2500/1:5000, anti-mouse-HRP (Sigma Aldrich): 1:5000, anti-rabbit-HRP (Sigma Aldrich): 1:5000

#### Immunofluorescence

5x10^4^ HeLa TRIM21 KO cells were plated on cover slips (Corning BioCoat, Poly-D-Lysine, 12 mm) in 24-well plates and allowed to adhere over night. After treatment, cells were washed three times with PBS, fixed in 4% PFA (Thermo Fisher Scientific), permeabilized in 0.1% Triton X-100 in PBS (PBST) and blocked with 5% BSA (Thermo Fisher Scientific) in PBST. The antibodies were also diluted in 5% BSA-PBST. The primary antibody was incubated over night at 4°C, the secondary antibody was incubated for 1h at room temperature. Antibody staining was performed in the 24-well plate, diluting the antibody as specified in 200 μL 5% BSA-PBST. If scarce antibody was used, the staining was performed by inverting the coverslip onto a 30 μL droplet of the diluted antibody on parafilm and incubating it in a box containing wet tissue paper. After each antibody incubation, cells were washed four times with PBS. The last wash after secondary antibody incubation was made into water. The cover slips were carefully dried and inverted onto DAPI containing hardset-mounting medium (Vectashield).

##### Protein VI Staining

1˚: anti-pVI (a kind gift from Urs Greber (Institute of Molecular Life Sciences, Zurich, Switzerland)), affinity purified rabbit polyclonal, 1:8, staining technique: cover slip inversion). 2˚: anti-rabbit-AF647 (Thermo Fisher, 1:500).

##### Galectin 3 Staining

anti-Galectin3- PE (eBioscience (M3/38), 1 μg/mL)

##### Virus Staining

1°: recombinant human 9C12 (100 ng/mL), 2°: anti-human IgG Fab-FITC (Sigma Aldrich (F5512), 1:200)

##### Virus + 9C12 Staining

1˚: anti-human IgG Fab-FITC (Sigma Aldrich (F5512), 1:200), anti-human IgG Fab (Sigma Aldrich (I5260), 1:200). 2˚: anti-goat-AF647 (ThermoFisher (A21447), 1:1000).

##### Lamin B1 Staining

1˚: anti-Lamin B1 (Abcam (ab16048), 1:1000). 2˚: anti-rabbit-AF568 (ThermoFisher (A-11011), 1:1000)

#### Flow Cytometry

All FACS assays were performed in 96-well round bottom plates (Corning). For neutralization assays, cells were harvested by trypsinization. For cell surface staining, cells were harvested by scraping at the indicated time points and fixed in 4% PFA (Thermo Fisher Scientific). Surface bound virus and 9C12 was detected using anti-human IgG-AF568 (Thermo Fisher, 1:1000). FcγRs were detected using anti-CD64-FITC (10.1, eBioscience, 0.2μg/sample), anti-CD32-PE (6C4, eBioscience, 25 ng/sample) and anti-CD16-APC, eBioCB16, eBioscience, 12 ng/sample). After antibody incubation, cells were washed three times with PBS-FBS and resuspended in 100 μL PBS-FCS.

Samples were acquired on a BD LSRII or a BD LSRFortessa flow cytometer (BD Biosciences).

For staining of whole blood, red blood cells were lysed using red blood cell lysis buffer (Miltenyi). After Fc-block (93, eBioscience, 1:100) white blood cells were stained with α-CD3 (17A2, BioLegend, 2.5 μg/mL), α-CD8a (KT15, BioRad, 0.125 μg/mL), H-2K^b^-SIINFEKL tetramer (MBL International, 2 μL/sample) and viability dye (1:2500, eBioscience). Samples were acquired on an iCyt Eclipse flow cytometer (Sony Biotechnology).

All flow cytometry data was analyzed using FlowJo software (FlowJo, LLC).

#### qPCR

RNA from mouse liver was isolated using the RNeasy lipid tissue mini kit (Qiagen) and RNA from cells was extracted using an RNeasy Mini Kit (Quiagen), including on column gDNA digestion. RNA was reverse transcribed using SuperRT (Cambio) according to manufacturer’s instructions. The qPCR reactions were set up using 5 μL Taqman fast master mix (Thermo Fisher Scientific), 0.5 μL Taqman primer (Thermo Fisher Scientific) and the desired amount of cDNA in a final volume of 10 μL. The reactions were assembled in a MicroAmp Optical 96-Well Reaction Plate (Thermo Fisher) and acquired on a StepOne Real-Time PCR System (Thermo Fisher Scientific).

##### Taqman Primers (Thermo Fisher Scientific)

Actb: Mm00607939_s1, Clec4f: Mm00443934_m1

##### Taqman Primers (Custom)

Ad5 for: TTGCGTCGGTGTTGGAGA, Ad5 rev: AGGCCAAGATCGTGAAGAACC, Ad5 probe: FAM-CTGCACCACATTTCGGCCCCAC-TAMRA

#### Elisa

9C12 variants were captured in 96-well EIA/RIA 3590 plates (Corning Costar) coated with Ad5 derived hexon protein (BioRad, #MPP002). 1μg/ml hexon diluted in PBS and 1000.0–0.45 nM 9C12 variants was used for the TRIM21 Elisa while 5μg/ml hexon and 6000.0-93.75 nM 9C12 was used for the C1q Elisa. PBS containing 0.05% Tween 20 (Sigma Aldrich) PBS/T was used as washing buffer between each layer, while PBS/T containing 4% skimmed milk powder (Acumedia) (PBS/T/S) was used as blocking and dilution buffer. Binding of TRIM21 containing an N-terminal His_6_-lipoyl tag (Clift et al., 2017) (1 μg/ml) was detected using an AP-conjugated anti-His_6_ antibody from mouse (Abcam, #ab49746) (diluted 1:5000). Binding of human C1q (Complement Technology) (1 μg/ml) was detected using a primary rabbit anti-hC1q antibody (DAKO, #A0136) and a secondary HRP-conjugated anti-rabbit IgG Ab from donkey (GE Healthcare) diluted 1:10.000 and 1:5000, respectively. Binding of TRIM21 to C1q occupied 9C12 variants was performed by capturing the Abs on 5μg/ml hexon followed by addition of a 2-fold molar excess of C1q before addition of 1 μg/ml TRIM21 and detection as above. Binding was visualized using AP substrate (Sigma-Aldrich) or TMB solution (CalBiochem) and the HRP-TMB enzymatic reaction was terminated by addition of 1 M HCl. The 405 nm (AP) and 450 nm (HRP-HCl) absorption spectra were acquired using a Sunrise plate reader (TECAN). The binding responses in the C1q Elisa were background subtracted using the values from wells without 9C12.

For quantification of Ad5-specific antibodies in NHS, 100 μl Ad5-GFP (5.0 x10^7^ PFU/ml) were coated in 96-well EIA/RIA plates (CorningCostar) and blocked with PBS/S. The plates were washed four times with PBS/T before titrated amounts of WT 9C12 (1000.0 – 0.45 ng/ml or 1000.0 – 10.0 ng/ml) and NHS (Complement Technology) (1:400 – 1:874800 or 1:100 – 1:10.000) diluted in PBS/T were added and incubated for 1 hour at RT. Bound antibodies were detected using an AP-conjugated anti-human IgG (Fc-specific) antibody from goat (Sigma Aldrich) and visualized by addition of phosphatase substrate (Sigma Aldrich). The 405nm absorbance spectrum were measured using a Sunrise plate reader (TECAN).

### Quantification and Statistical Analysis

#### Statistical Analysis

Unless otherwise indicated, statistical analyses were performed using GraphPad Prism 7 software (GraphPad). Error bars depict the SD or SEM as indicated. Data was considered to be statistically significant when p < 0.05 by two-tailed Student’s t test. In figures, asterisks denote statistical significance as calculated using a Student’s t test (^∗^, p < 0.05; ^∗∗^, p < 0.01; ^∗∗∗^, p < 0.001; ^∗∗∗∗^, p < 0.0001). As indicated in the relevant figures (Figures 4 and 5), the term ‘n’ refers to the number of cells used in the analysis.

## Supporting Citations

The following reference appears in the Supplemental Information: Law and Dodds (1997).

## Supplemental Information

Document S1. Figures S1–S6

Document S2. Article plus Supplemental Information
