# Supplementary material for: Complement C4 Prevents Viral Infection through Capsid Inactivation
Source: Cell Host Microbe. 2019 Apr 10;25(4):617–629.e7. doi: 10.1016/j.chom.2019.02.016 (PMC6461443; doi:10.1016/j.chom.2019.02.016)
Supplement: Document S1. Figures S1–S6 [file mmc1.pdf]

Cell Host & Microbe, Volume *25*

Supplemental Information

Complement C4 Prevents Viral Infection through Capsid Inactivation

Maria Bottermann, Stian Foss, Sarah L. Caddy, Dean Clift, Laurens M. van Tienen, Marina Vaysburd, James Cruickshank, Kevin O'Connell, Jessica Clark, Keith Mayes, Katie Higginson, Heidrun E. Lode, Martin B. McAdam, Inger Sandlie, Jan Terje Andersen, and Leo C. James

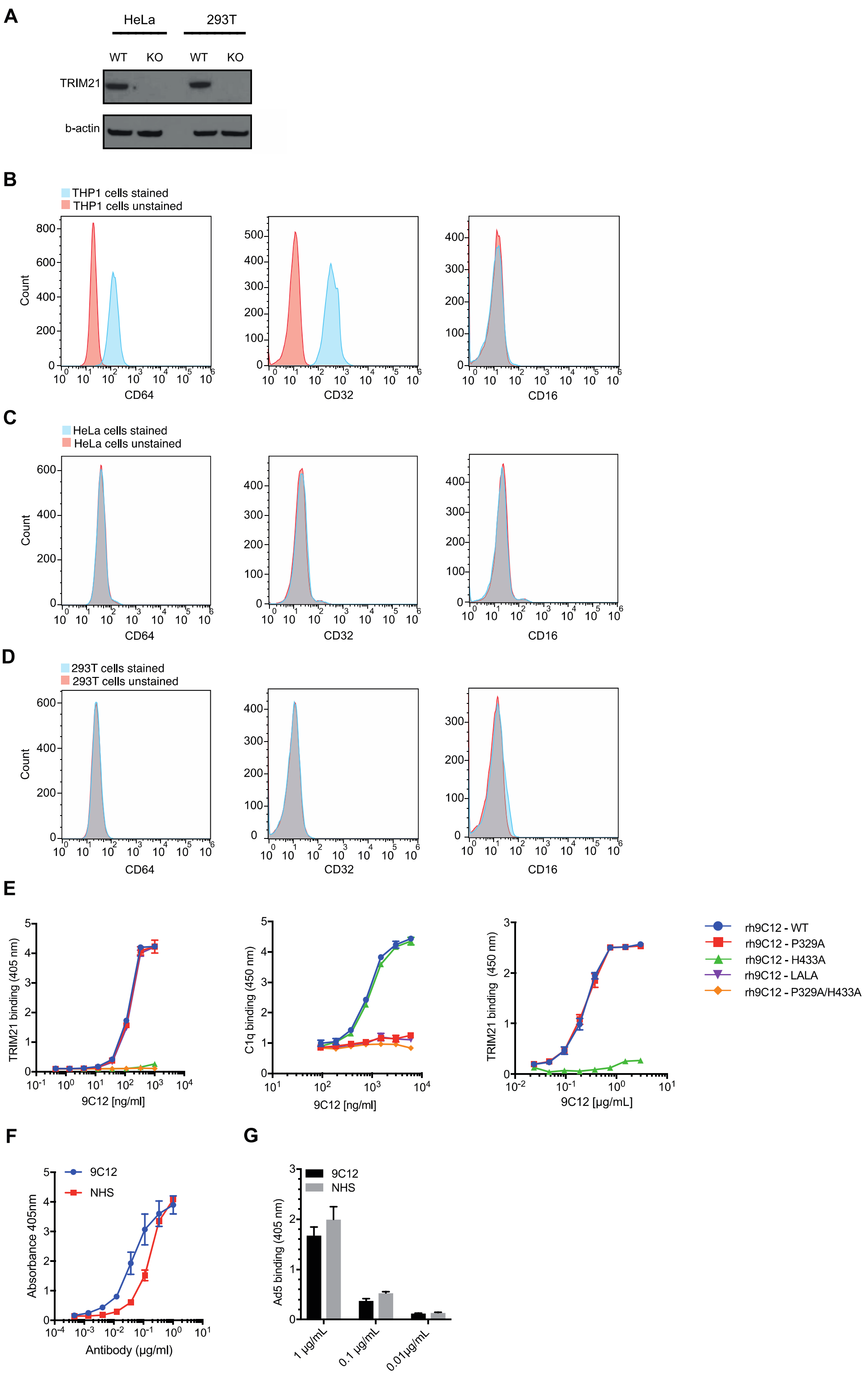

Figure S1: Binding of 9C12 variants to C1q and TRIM21, Related to Figure 1.
(**A**) Western blot showing TRIM21 KO in 293T and HeLa cells (generated by CRISPR/Cas9). (**B-D**) Staining for FcγRI (CD64), FcγRII (CD32) and FcγRIII (CD16) on THP-1 cells (B), HeLa cells (C) and 293T cells (D). THP-1 cells serve as a positive control for the expression of CD64 and CD32. (**E**) ELISA showing binding of 9C12 variants to TRIM21 (left) and C1q (middle) and binding of TRIM21 to C1q occupied 9C12 (right). Error bars depict +/- SD of duplicates from one representative experiment. (**F**) Titration of NHS against 9C12 to determine the Ad5-specific antibody concentration in NHS as 97.2µg/mL. (**G**) Detection of the amount of antibody bound to virus when 9C12 was used at 1µg/mL - 0.001µg/mL and NHS was diluted 1:100 – 1:10000.
Original western blots are included in Figure S6.

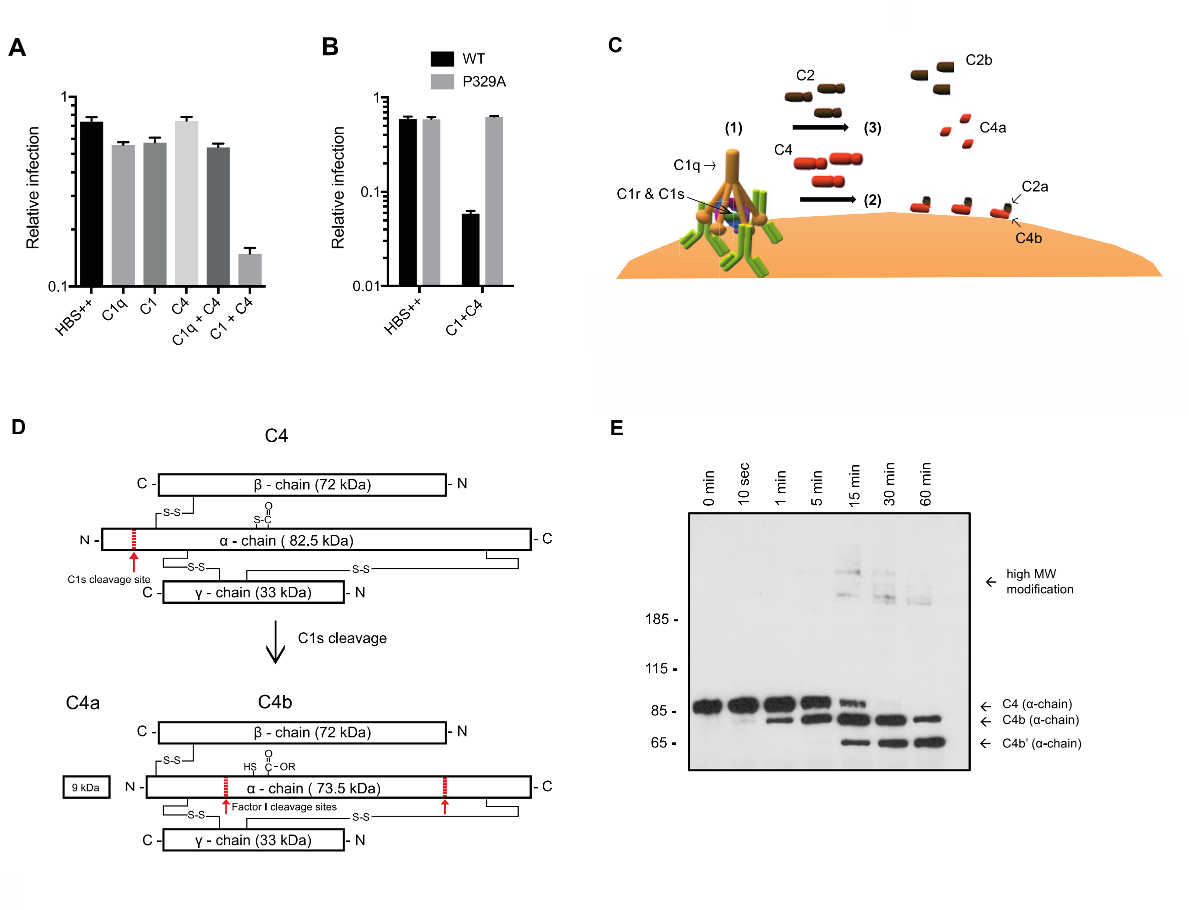

Figure S2: C1 and C4 mediate neutralization of Ad5, Related to Figure 2.
(**A**) Neutralization of Ad5 in HeLa TRIM21 KO cells using 9C12-WT in presence of the indicated proteins. (**B**) Neutralization of Ad5 in HeLa TRIM21 KO cells using 9C12-WT and P329A in the presence of buffer (HBS++) or C1/C4. (**C**) Cartoon of the early steps of the complement cascade. C1q binds to antibody, which activates the proteases of the C1 complex, C1r and C1s. C1s cleaves C4 into C4a and C4b. This exposes a highly reactive thioester in C4b, which attaches to nearby hydroxyl and amino groups. C1s also cleaves C2, and C2a associates with C4b forming the C3 convertase. (**D**) Schematic of C4 protein with cleavage sites for C1s and Factor I shown in red. Adapted from (Law & Dodds 1997). (**E**) Western blot of C4 (α-chain) cleavage in NHS (top) or C1q depleted serum (bottom) in the presence of Ad5 and 9C12-WT over 60 min (long exposure).
Original western blots are included in Figure S6.

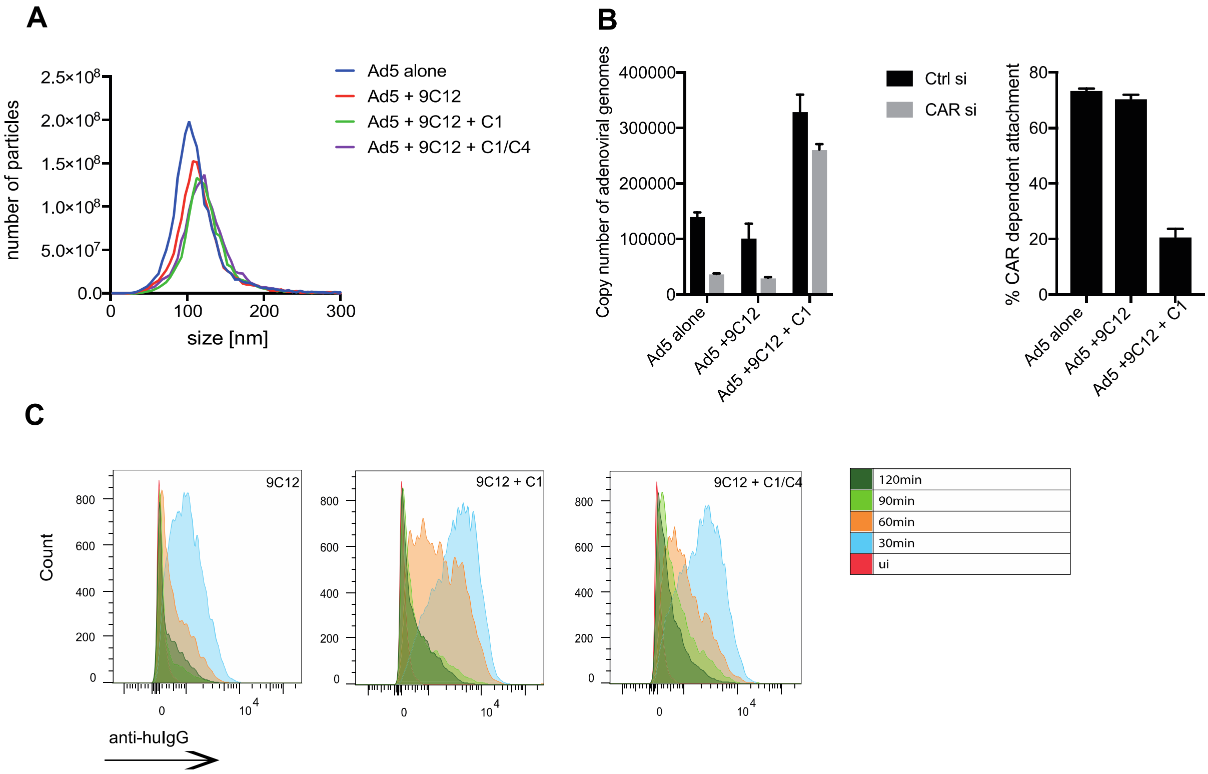

Figure S3: C1 and C4 neither mediate viral particle aggregation nor prevent their internalization, Related to Figure 3.

(**A**) The size of Ad5 particles acquired using nanoparticle tracking analysis. Sizes were determined for Ad5 alone, Ad5+9C12-WT, Ad5+9C12-WT+C1 and Ad5+9C12-WT+C1/C4. Slight increases in particle size upon addition of 9C12 and C1 likely reflect an increase in particle circumference, not formation of immune complexes. (**B**) Ad5 copy number after 30 min of continuous infection in HeLa TRIM21 KO cells using Ctrl or CAR siRNA. The viral copy number was determined by qPCR (left). CAR dependent attachment was determined as the ratio of viral copies in siCtrl cells to viral copies in siCAR cells (right). (**C**) Histograms of the internalization assay shown in Figure 3D.

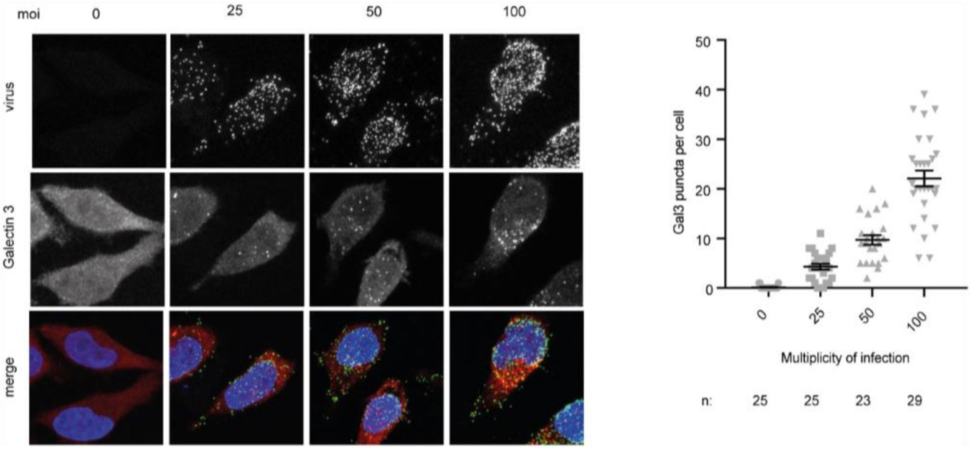

Figure S4: Galectin 3 staining increases proportionally to virus multiplicity of infection, Related to Figure 4. HeLa TRIM21 KO cells were infected with the indicated multiplicity of infection (moi) of Ad5. Cells were harvested 30 min post infection and stained with an anti-Galectin 3 antibody. Left: Virus staining is depicted in green, Galectin 3 staining is depicted in red. The images were acquired using a 63x objective. Right: Quantification of Galectin 3 puncta per cell in the indicated conditions. Error bars show mean +/- SEM of the indicated number of cells (n).

A

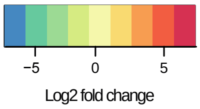

Log2 fold change	Gene symbol	FDR adj. P-value
- 5.39	*C1qa*	1.49E-53
- 4.53	*C1qb*	9.02E-30
- 4.31	*C1qc*	2.34E-25
- 7.19	*Vsig4*	3.65E-11
2.46	*Itgam*	0.0019
- 1.53	*C3ar1*	0.0167
0.41	*Mbl1*	0.0742
0.38	*Serping1*	0.119
0.39	*Cfb*	0.174
0.46	*C4b*	0.194
0.42	*Cfh*	0.236
0.30	*Hc*	0.239
- 0.69	*Cd46*	0.517
0.18	*Clu*	0.533
4.31	*C1s2*	NA
0.37	*C3*	0.581
0.18	*Vtn*	0.602
- 2.19	*C1rb*	0.664
0.23	*Masp1*	0.667
0.18	*C4bp*	0.778
- 1.04	*C5ar1*	0.778
0.20	*Mbl2*	0.788
0.20	*C8g*	0.795
2.67	*Cfd*	NA
0.87	*Itgax*	0.813
- 0.30	*Itgb2*	0.847
- 0.08	*C1s1*	0.884
0.06	*C1ra*	0.917
- 0.16	*C8b*	0.922
- 0.10	*Cd59a*	0.930
- 0.70	*Cr2*	0.940
0.19	*C8a*	0.945
0.05	*Cfi*	0.950
0.04	*Masp2*	0.962
- 0.24	*Cd59b*	0.966
0.05	*Cd55*	0.969
0.11	*C6*	0.988
- 0.12	*C7*	0.990
- 0.20	*Cd55b*	NA
- 0.03	*C9*	0.996
9.61E-06	*C2*	0.999
- 2.01	*Itgb2l*	NA
NA	*Zp3r*	NA

B

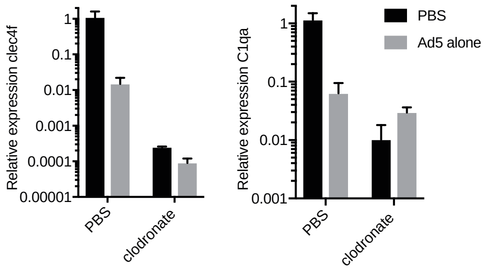

Figure S5: C1q transcripts become downregulated during Ad5 infection, Related to Figure 6.
(**A**) Table of genes of the complement pathway (Kegg pathway map 04610) comparing log2 fold changes between uninfected mice and mice infected with Ad5 (GSE119119). (**B**) Expression levels of the Kupffer cell marker *clec4f* (left) and *C1qa* (right) in mice after 48h after treatment with clodronate liposomes and 4h post infection with Ad5.

Figure 1D

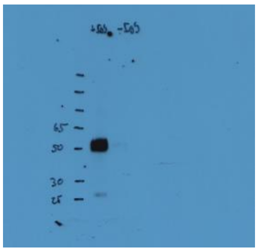

Figure 2A

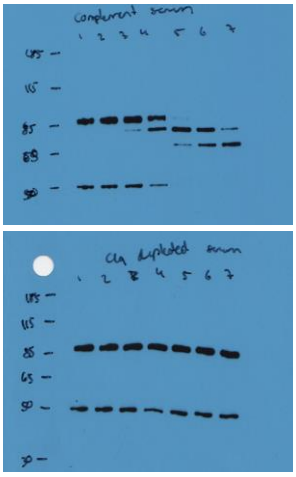

Figure 2C

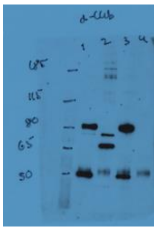

Figure 3B

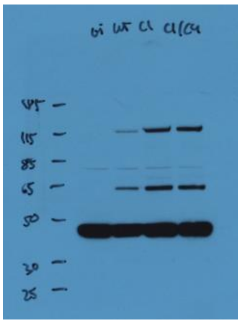

Figure 4A

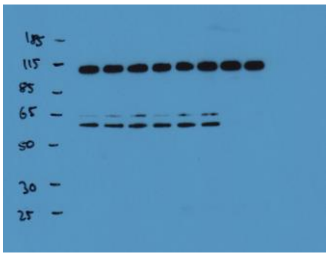

Figure 4B

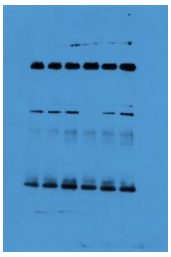

Figure S1A

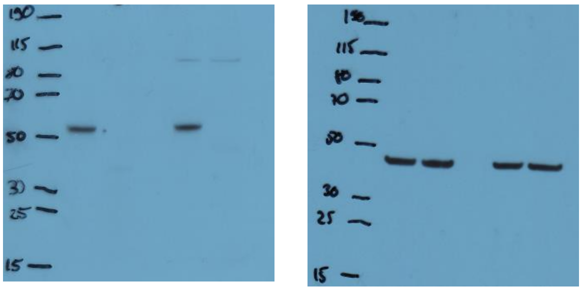

Figure S2E

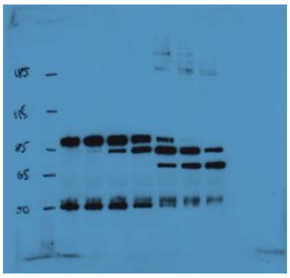

Figure S6: Original western blots of the indicated main figures, Related to Figures 1, 2, 4, S1 & S2. These are complete western blots for the indicated figures. Molecular weight markers are given at the side.
